# Stakeholder insights into hospital-home transitions for older adults in a decentralised health system: a qualitative study

**DOI:** 10.1186/s12913-026-14662-4

**Published:** 2026-05-21

**Authors:** Laura Maria Steiner, Sandra M. G. Zwakhalen, Loris Bonetti, Alissa Borner, Agata Ferrari, Sabine Hahn

**Affiliations:** 1 Nursing Research Competence Centre, Department of Nursing, Ente Ospedaliero Cantonale, Bellinzona, Switzerland; 2 Department of Business Economics, Health and Social Care, University of Applied Sciences and Arts of Southern Switzerland, Manno, Switzerland; 3 Department of Health Services Research, Care and Public Health Research Institute CAPHRI, Maastricht University, Maastricht, Netherlands; 4 Department of Nursing, Beata Vergine Hospital, Ente Ospedaliero Cantonale, Mendrisio, Switzerland; 5 School of Health Professions, Applied Research & Development in Nursing, Bern University of Applied Sciences, Bern, Switzerland

**Keywords:** Transitional care, Hospital discharge, Older adults, Decentralised health systems, Continuity of care, Qualitative research, Care transitions

## Abstract

**Background:**

Hospital-home transitions are critical points in the care trajectory for older adults, requiring alignment between clinical decision-making, communication, and home-based support to maintain care continuity. In decentralised health systems, where responsibilities are distributed across multiple providers, these transitions may be vulnerable to fragmentation. Although transitional care has been studied, little is known about how it unfolds in decentralised, mixed-provider systems, where accountability is distributed across providers. This study constitutes the contextual inquiry phase of a Medical Research Council-guided intervention development programme and explores how hospital-home transitions are experienced and enacted in a decentralised Swiss region.

**Methods:**

We conducted a qualitative descriptive study in southern Switzerland involving 26 participants. Data were generated through two focus groups with hospital–based professionals (*n* = 18) and seven semistructured interviews with community stakeholders. These included general practitioners, public and private home-care providers, self-employed nurses, one patient, and one family caregiver. Topic guides were informed by international discharge standards and Transitions theory constructs. Audio recordings were transcribed verbatim and analysed via reflexive thematic analysis, combining inductive coding with deductive sensitising concepts from Transitions theory. Reporting follows COREQ.

**Results:**

Five interrelated themes described hospital-home transitions as fragile and often sustained through ad hoc support: (1) fragile communication pathways marked by inconsistent and person-dependent information flows; (2) medication safety as a systemic fault line, with frequent discrepancies between hospital and home regimens; (3) diffuse accountability and uneven bridging roles, whereby coordination relied on discretionary efforts; (4) families compensating for coordination gaps; and (5) practices that supported smoother transitions, including early discharge planning, proactive liaison roles, and structured information tools.

**Conclusions:**

Across stakeholder groups, continuity of care was maintained through informal coordination by professionals and families. Strengthening hospital-home transitions may require more reliable discharge communication routines, clearer medication processes, and more structured involvement of family caregivers and community providers. These findings, drawn mainly from professional perspectives, can inform the co-design of transitional care improvements tailored to the local context.

**Trial registration:**

Not applicable.

**Supplementary information:**

The online version contains supplementary material available at 10.1186/s12913-026-14662-4.

## Background

Transitions between care settings are widely recognised as vulnerable periods, particularly for older adults living with multimorbidity [1–3]. Hospital discharge requires alignment between clinical decisions, effective communication, and appropriate home-based support to maintain care continuity [4]. When this alignment is incomplete, patients may experience medication errors, unmet care needs, avoidable emergency visits, and hospital readmissions [2, 5–8]. Transitional care, understood as the set of coordinated actions that support continuity across settings, therefore plays a central role in patient safety and care quality [4, 9].

Transitions theory provides a conceptual lens for understanding why hospital‒home transitions constitute a particularly fragile process [6, 10]. The theory conceptualises the move from hospital to home as a boundary crossing experience [5, 11] shaped by contextual factors such as patients’ health status, the availability of family caregivers, and structures supporting preparation and coordination across settings [11]. It emphasises the importance of role clarity, anticipatory preparation, and effective communication in supporting safe transitions [5, 10, 11]. When these conditions are weak, patients and family caregivers may feel uncertain or unprepared for life at home [5, 6]. Although numerous transitional care interventions, such as structured discharge planning, medication reconciliation, and early follow-up, have been developed and evaluated, their effects remain variable [7, 12]. Persistent reports of communication breakdowns and fragmented workflows suggest that transitional care involves more than a series of clinical tasks. It is also shaped by how organisations work together and how care relationships across settings are coordinated.

These challenges are particularly salient in decentralised health systems, where responsibility for organising and financing care is distributed across regional or local authorities and delivered by multiple autonomous providers [13, 14]. In such systems, authority is located primarily at subnational levels [14]. Decentralised arrangements are found in several European countries (e.g., Spain, Italy, Belgium), whereas other countries, such as the United Kingdom (UK), have been described as having more centralised features of governance [4, 10]. While decentralisation may enhance responsiveness to local needs, it can also contribute to variation in coordination practices and information flows across care settings [15, 16].

In Switzerland, healthcare is organised at the cantonal level (cantons being the 26 member states of the Swiss Confederation, each with substantial autonomy over health policy, hospital planning, and the regulation of care delivery) and is delivered through a complex mix of public hospitals, private clinics, community agencies, and self-employed providers [13, 15]. This structural diversity is associated with variable discharge routines, limited digital interoperability, and delays in the transmission of information to general practitioners (GPs) and home-care services [13, 14]. Such conditions may shape how hospital‒home transitions are organised and experienced in everyday practice. However, similar coordination challenges have also been documented in more centralised systems, suggesting that these difficulties extend beyond formally decentralised contexts [4].

Southern Switzerland provides a particularly illustrative case for examining these dynamics [14, 17]. The region is characterised by an ageing population and a heterogeneous provider landscape spanning the public and private sectors [14, 18]. Although bridging roles such as advanced practice nurses (APNs) or liaison nurses exist in some organisations, they are not consistently embedded across settings [19]. As a result, coordination is often carried out in an ad hoc manner, relying on local routines, interpersonal relationships, and individual initiative [19]. Families frequently take on coordination tasks to compensate for system gaps, including managing appointments, clarifying care plans, and supporting continuity after discharge. This reliance on informal coordination effort points to recognised limits in current transitional care arrangements in decentralised systems [5, 20, 21].

Despite the growing international literature on transitional care [22], evidence on how transitions are enacted within highly decentralised, mixed-provider systems remains relatively limited [23]. In this study, mixed-provider refers to care delivered across public, private, and self-employed health providers operating within multiple organisations. System refers to the wider cantonal care landscape and its organisational interfaces, and organisational refers to routines at the level of individual hospitals or providers. Research that integrates the perspectives of hospital clinicians, GPs, community-based professionals working in both public and private organisations, self-employed nurses, patients, and family caregivers across these contexts remains limited [10, 24]. Understanding how these diverse stakeholders experience hospital‒home transitions, and which factors they perceive as shaping those experiences, is essential for informing contextually appropriate service improvements [6, 8].

The aim of this study was therefore to explore how diverse stakeholders experience hospital-home transitions for older adults in a decentralised Swiss canton, with a focus on identifying perceived barriers and enablers [25]. This work constitutes the contextual inquiry phase of a broader intervention-development programme guided by the Medical Research Council (MRC) framework [25]. The findings are intended to inform the co-design of transitional care improvements tailored to the local context.

## Methods

### Study design

We conducted a qualitative descriptive study to explore how patients, family caregivers, and healthcare professionals experience hospital‒home transitions for older adults in a decentralised health system. Although framed as qualitative descriptive to reflect its pragmatic, practice-oriented aim, it was informed by an interpretivist stance, while participants’ accounts were approached as shaped by their social and professional positions. This orientation shaped both data generation (e.g., using open-ended questions) and analysis (e.g., attending to how participants attributed meaning to transitional events). We employed reflexive thematic analysis [26–28] and generated data through focus groups and semistructured interviews. Transitions theory served as a sensitising framework [11]. Its constructs (e.g., preparation, role clarity, and environmental conditions) informed deductive coding, while the broader orientation to boundary-crossing shaped the interpretation of participants’ accounts. More specifically, statements concerning readiness for discharge, anticipatory planning, and preparedness for care at home were coded as relating to preparation, statements concerning uncertainty over who should notify, coordinate, prescribe, reconcile, or follow up were coded as relating to role clarity, and statements concerning discharge timing, weekend discharges, digital interoperability, service availability, and family support were coded as relating to contextual or environmental conditions. These constructs guided the analysis. Reporting follows the Consolidated Criteria for Reporting Qualitative Research (COREQ) [29].

### Setting

The study was conducted in southern Switzerland, an Italian-speaking region with approximately 350,000 residents [18, 30]. Hospital-home transitions in this region unfold within a highly decentralised and mixed-provider landscape. Acute hospital care is delivered through the public hospital network, Ente Ospedaliero Cantonale (EOC), which operates a multisite system comprising eight hospital and rehabilitation sites and approximately 850 acute care beds [31]. In parallel, several sizeable private clinics provide acute and specialised services (around 500 beds) [32].

Community-based care is delivered by a large and diverse set of providers. As of July 2025, the cantonal register listed 79 authorised home-care organisations, of which six were publicly organised services [33], alongside a substantial private sector. Community care is further complemented by self-employed nurses (261 listed on the cantonal register as of May 2025, among those who consented to public listing) [33] and an estimated 266 full-time equivalent (FTE) primary care physicians, including GPs, corresponding to 0.76 FTE per 1,000 inhabitants [34].

In Switzerland, home care includes nursing, assistance, and supportive services delivered in a person’s own home, typically for older adults, individuals with chronic illness or disability, or those following hospital discharge. Home care covers both medical and nursing tasks and support with activities of daily living and is distinct from institutional long-term care such as nursing homes [35]. Access to reimbursed services generally requires a physician’s prescription, with costs distributed through compulsory health insurance, patient co-payments, and cantonal or municipal subsidies [35]. Home-care organisations and self-employed nurses therefore play a central role in supporting older adults in the period after discharge [35]. This decentralised, mixed-provider configuration reflects the structural complexity that characterises hospital-home transitions for older adults in this setting.

### Participants and sampling strategy

Purposive sampling was used to achieve maximum variation across professional roles and care settings involved in hospital‒home transitions for older adults. Sampling decisions were guided by the study aim and sought to capture diverse perspectives across organisational boundaries. Within the hospital setting, participants were selected to represent professions directly involved in discharge decision-making and coordination, including physicians, nurses, pharmacists, social workers, and managerial leaders. In the community setting, sampling sought variation across care delivery models and professional roles, including GPs, public and private home-care nurses, and self-employed nurses. To include experiential perspectives on recent transitions, a small number of patients and family caregivers were purposively included. Patients were eligible if they had been discharged within the previous eight weeks, a timeframe chosen to support accurate recall while allowing sufficient time for reflection.

Recruitment proceeded iteratively within predefined role categories, with ongoing assessment of conceptual coverage as data collection progressed. Sample adequacy was assessed using Malterud’s information-power model, which proposes that smaller samples may be sufficient when the study aim is focused, the sample is specific, dialogue quality is high, and analysis is in-depth [36]. Information power was judged to be sufficient for the professional sample because the study aim was relatively focused (hospital-home transitions for older adults in one canton), participants occupied specific roles at the hospital–community interface, and the data generated were rich enough to support in-depth and theory-informed analysis [36]. Information power was more limited in respect of experiential perspectives, as only one patient and one family caregiver participated. Accordingly, claims about patient and caregiver experience are interpreted cautiously.

The original sampling framework aimed to include multiple patients and family caregivers. However, recruitment in the immediate post-discharge period proved difficult, as older adults and family members were often physically unwell, emotionally burdened, or reluctant to participate. Consequently, only one patient and one family caregiver were enrolled.

### Recruitment and sample size

Twenty hospital staff members were initially invited to participate in focus groups by the authors (LMS, AB, AF) in coordination with departmental leadership, via personalised email invitations describing the study aim, eligibility criteria, and ethical approvals. Two invitees were unavailable and were replaced by colleagues in comparable roles. Two further invitees did not respond. The final hospital sample therefore comprised 18 participants. Community stakeholders were recruited via email invitations sent to three GPs, two public home-care nurses, three private home-care providers, and two self-employed nurses. Patients and caregivers were approached in person by a trained researcher, provided with study information, and subsequently recontacted to confirm participation. Seven semistructured interviews were conducted with eight community stakeholder participants, including one conducted jointly with two participants who worked together in the same organisation and had requested a joint interview. This was considered appropriate given their shared role in transitional care. Across all data sources, the final sample comprised 26 participants.

### Data collection

Data were collected between May and September 2025 in Italian, the primary language spoken in the study region.

#### Focus groups

Two focus groups were conducted in person at the hospital. The first included participants in coordination and managerial roles. The second involved frontline professionals directly involved in discharge processes. Each focus group lasted approximately 90 min. Discussions were facilitated by an experienced qualitative researcher, a second researcher took field notes noting contextual and non-verbal observations.

#### Interviews

Seven semistructured interviews were conducted with community stakeholders. Six interviews took place in person, either at the hospital or in the participants’ homes, and one interview was conducted online. The interviews lasted between 30 and 70 min and were conducted in a private setting.

#### Focus group and interview guides

Topic guides were developed following Kallio et al.’s systematic approach for semistructured interviews [37]. The content was informed by two international discharge standards: the National Institute for Health and Care Excellence (NICE) guideline NG 27 on transitions between inpatient hospital settings and community or care home settings for adults, which addresses communication, medication safety, and the involvement of patients and carers during transition, and the German Network for Quality Development in Nursing (DNQP) discharge standard, which covers assessment, planning, and coordination of hospital discharge [38, 39]. Core domains from these standards were cross-referenced with Transitions theory constructs (preparation, role clarity, environmental conditions) to ensure that topic guides addressed both operational processes and the experiential dimensions of transitions.

The initial topic domains included communication processes, role clarity, continuity of care, preparation of patients and family caregivers, and perceived opportunities for improvement. Guides were piloted with one hospital nurse and refined iteratively. As data collection progressed, additional topics were incorporated, including medication reconciliation, digital interoperability, and structured follow-up after discharge. Ten core questions with optional probes were used flexibly across interviews and focus groups [38, 39] (see Additional file 1).

All sessions were audio-recorded and transcribed verbatim. Field notes were used to document contextual details. All transcripts were pseudonymised and stored securely.

### Data analysis

The data were analysed using Braun & Clarke’s reflexive thematic analysis [26–28]. The first author read all transcripts repeatedly to achieve immersion in the data and conducted initial coding using MAXQDA (Version 24.10.0). Coding combined inductive attention to participants’ accounts with deductive sensitising concepts informed by Transitions theory. Semantic codes captured explicit descriptions of discharge practices, while latent codes captured underlying organisational and relational dynamics. To support analytic dialogue and encourage discussion about coding decisions, two transcripts were independently coded by a second researcher [26–28]. Coding decisions and emerging interpretations were discussed within the research team. Themes were developed and refined through team discussions until they adequately captured the main patterns in the dataset and held together analytically. We used thematic sufficiency rather than data saturation to judge when analysis was complete [36]. Quotations were translated from Italian into English using DeepL and subsequently checked by a bilingual researcher to preserve accuracy and meaning. Although Transitions theory informed analytic attention, theme development remained inductive and reflexive, which is consistent with the study’s interpretivist orientation. A thematic map was developed to visualise relationships between themes and subthemes [40], and a cross-theme synthesis was subsequently conducted to examine how the themes interacted and collectively shaped understanding of hospital-home transitions.

### Research team and reflexivity

The multidisciplinary research team brought expertise in nursing science, health services research, and clinical practice. Data collection was conducted by three researchers (LMS, AB, and AF), all of whom held Master of Science degrees in Nursing and had been trained in qualitative interviewing. The first author had professional familiarity with some hospital participants but held no supervisory or therapeutic relationships with them. To address the potential influence of this familiarity, coding from early transcripts involving these participants was compared with coding from transcripts involving participants with whom there was no prior relationship. These comparisons were discussed in analytic meetings to identify potential blind spots or unexamined assumptions. The other two interviewers had no prior relationships with the participants. Reflexivity was supported through memo writing, team dialogue, and critical debriefing with senior researchers (SZ, SH, LB), whose contributions focused on methodological and conceptual oversight. The team’s professional interest in transitional care was acknowledged as both a potential influence on interpretation and a resource for analytic insight.

### Trustworthiness

Rigour was supported with reference to Lincoln and Guba’s criteria for trustworthiness [41]. Credibility was enhanced through triangulation across stakeholder groups and data sources. Dependability was strengthened through an audit trail documenting analytic decisions and reflexive memos. Confirmability was supported through reflexive journaling and external peer debriefing. Transferability was facilitated by a detailed description of the study context, participants, and the transitional care setting. Participants did not review transcripts or findings, which is consistent with the interpretivist stance and the analytic aim of reflexive thematic analysis.

### Ethical considerations

All procedures were carried out in accordance with the Declaration of Helsinki (1964) and its subsequent amendments. Ethical approval was obtained from the Cantonal Ethics Committee (Protocol 2025-00025, Ref. CE 475). All participants provided written and verbal informed consent prior to participation, including consent for audio-recording. Anonymity and confidentiality were ensured through pseudonymisation and removal of identifying details.

## Results

The sample reflected a broad range of professional perspectives relevant to hospital‒home transitions, with experiential representation limited to one recently discharged patient and one family caregiver (Table 1).

**Table 1:** Participant characteristics

Stakeholder group	*N*	Gender (F/M, *n*)	Roles/positions	Setting
Hospital leadership/support (FG)	7	5/2	Nurses (senior leadership, ward and sector managers, clinical nurse specialist); pharmacist; lead physician	Hospital (public)
Hospital frontline (FG)	11	9/2	Nurses (medical/surgical; APN, case manager); physician and assistant physician; social worker; therapists (dietitian, physiotherapist)	Hospital (public)
Patient	1	1/0	Recently discharged patient	NA
Family caregiver	1	0/1	Son (family caregiver)	NA
GP	1	1/0	General practitioner	Community-based
Public home-care providers	1	0/1	Nurse manager/liaison nurse	Community-based
Private home-care providers	2	0/2	Nurse co-managers	Community-based
Self-employed providers	2	0/2	Community nurses	Community-based

Notes: F= Female; M= Male, n= number; FG = focus group; APN = advanced practice nurse; NA = not applicable

### Overview of themes

**Fig. 1:**
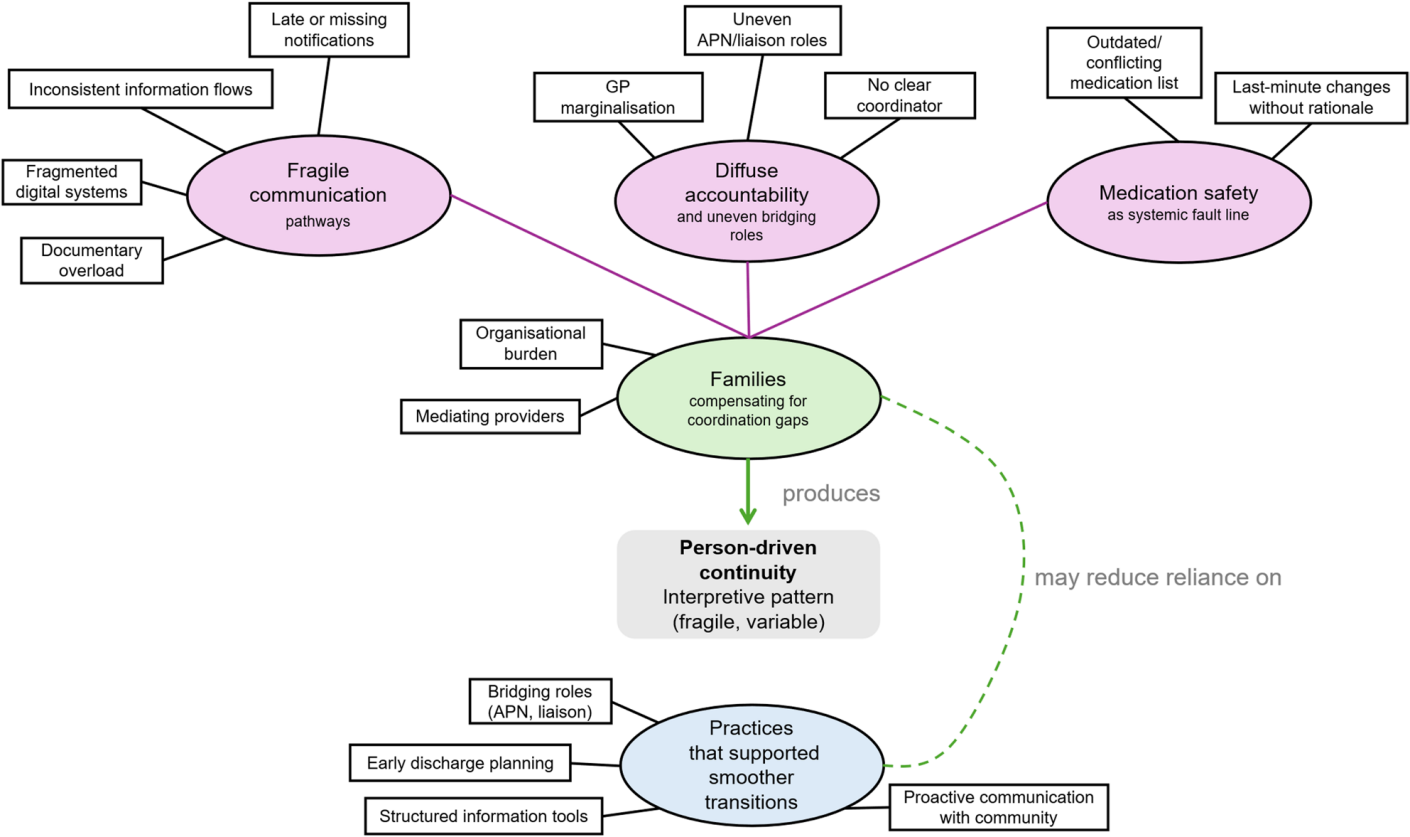
Thematic map of five themes and their relationship to the interpretive pattern of person-driven continuity. Themes 1–3 (purple) depict systemic conditions that create coordination demands. Theme 4 (green) represents compensatory work through which gaps in coordination are managed, giving rise to a pattern of person-driven continuity that is often fragile and variable. Theme 5 (blue) identifies organisational practices that may reduce reliance on individual effort and support more embedded forms of continuity. Spatial arrangement and connecting lines reflect conceptual relationships, not hierarchy. Notes: Themes are shown as ovals, sub-themes as rectangles, and the interpretive pattern as a rounded grey rectangle. Purple lines indicate systemic conditions generating coordination demands; the green arrow indicates emergence of the pattern; the green dashed line indicates reduced reliance

Five interrelated themes described how stakeholders experienced hospital-home transitions: (1) fragile communication pathways; (2) medication safety as a systemic fault line; (3) diffuse accountability and uneven bridging roles; (4) families compensating for coordination gaps; and (5) practices that supported smoother transitions. Across stakeholder groups, continuity of care was often maintained through additional coordination efforts by professionals and family caregivers in the context of variable system support. We describe this recurring pattern as person-driven continuity. Figure 1 presents the thematic map [40].

#### Theme 1: Fragile communication pathways

Across stakeholder groups, communication during hospital‒home transitions was characterised by fragmentation, inconsistency, and reliance on individual effort. Rather than following a stable, predictable process, discharge communication unfolded through ad hoc exchanges shaped by local routines and personal networks. Participants described how this instability generated persistent uncertainty and required compensatory work, that is, additional effort by professionals and families to bridge missing or unclear information.

Hospital professionals highlighted the number of actors involved in discharge as a central challenge to reliable information transfer:

At times, the transfer of information is still quite fragmented, because there are many actors involved. We often observe that problems arise from an incorrect or fragmented handover of information. One factor that complicates this is the “jungle” in the community (…) there are many different services and having to interface with so many of them makes information transfer more difficult. (Hospital leader)

Community providers frequently described receiving information late or not at all, sometimes only after the patient had already returned home:

Sometimes we only find out after the fact (…). Suddenly they tell us the patient is already home… but without any clinical information. There was no discharge summary, no therapy plan, no explanation of what changed during hospitalisation. So, we start care without knowing what actually happened. (Home-care nurse)

Participants did not describe any shared system that reliably notified community providers when a patient was being discharged. Instead, information was passed on through discharge summaries, phone calls, provider-specific documents, and sometimes through family members. Community providers also reported uncertainty about whether the information they had shared with the hospital was taken into account during discharge planning.

Families often acted as intermediaries, identifying inconsistencies and seeking clarification:

In the discharge paperwork, on one page it said the follow-up visit was at the Civico (hospital) in Lugano… but on another sheet it indicated Mendrisio. So what did I do? I called the doctor here… and she checked and called me back (…) it wasn’t Lugano, it was Mendrisio. (Family caregiver)

Participants also described documentary overload and nonstandardised formats as barriers to effective communication, particularly for community nurses working under time pressure and managing information from multiple sources.

Overall, communication continuity often depended on individuals stepping in to clarify, relay, or follow-up missing information. Although the study focused on discharge, some participants also pointed to information gaps in the opposite direction, from home to hospital. In these accounts, information about patients’ home context and existing support was not always clearly communicated or considered when hospitalisation occurred. Communication difficulties were therefore not confined to discharge.

#### Theme 2: Medication safety as a systemic fault line

Medication management emerged as a key area in which fragmentation across care settings became most visible and clinically consequential. Medication information was described as unstable, often arriving late or reflecting an outdated regimen and being unevenly shared across providers. Medication safety therefore depended heavily on anticipatory and corrective work by professionals. Hospital and community professionals linked medication-related risks to the late completion of discharge documentation:

Often the discharge letter is written on the same day the patient is discharged. This makes it difficult to have a final, updated medication regimen to organise everything in the community. (Hospital professional)

Discharges occurring close to or during weekends further amplified risk, particularly when access to pharmacies or prescribers was limited.

From the perspective of community providers, these timing pressures contributed to the perception that some discharges were rushed, with attention focused on resolving acute medical problems while broader conditions for safe medication management at home were insufficiently assessed:

Sometimes discharges happen over the weekend and are not always confirmed, so there is a lot of uncertainty. From our point of view, these are rather rushed discharges: maybe the acute problem is resolved, but everything else is not considered, like the support network or whether the person is actually able to manage at home. (Home-care nurse)

Beyond timing, medication safety was further undermined by the circulation of multiple, inconsistent medication lists across care settings. Community nurses described encountering patients who had passed through several institutions, each of which had generated a different version of the medication regimen:

This patient, for example, went through two (and a half) hospitals… and ended up with three different medication charts. (Home-care nurse)

As a result, community providers often assumed responsibility for reconstructing medication regimens by comparing documents and contacting prescribers:

In the end, maybe we are the only ones who truly know what the current therapy is. (Home-care nurse)

The GP also confirmed that medication changes were often communicated without sufficient explanation, requiring postdischarge interpretation and verification:

In the initial discharge letter there is no explanation for the changes in medication. You can see that the therapy has been modified, but not why. (GP)

The clinical consequences of these discontinuities were also noted by hospital professionals, who explicitly linked gaps in medication continuity to avoidable deterioration and readmissions:

Then they may come back with decompensation simply because they did not take the medication. (Hospital professional )

Overall, medication management represented a systemic fault line during care transitions, with discontinuities that repeatedly exposed patients to increased risk of readmission. Medication safety often depended on professionals noticing discrepancies, checking documents, and contacting one another to resolve them.

#### Theme 3: Diffuse accountability and uneven bridging roles

Responsibility for coordinating transitions shifted fluidly across professionals and settings. This diffuse accountability meant that transitions were more smoothly managed for some patients but not for others. In some wards, well-established bridging roles supported coordination. In others, continuity depended on chance or family initiative:

Often, I know when a patient is admitted, yes. However, for the discharge, no. Many times, I only determine afterwards, when the patient calls me and says, “Doctor, I’m back home.” And then I realise that the patient has been discharged, but I haven’t received any information, no discharge letter yet, nothing. (GP)

Self-employed and home-care nurses similarly reported late or absent notifications, even when they had longstanding involvement with the patient. The quality of transitional care therefore varied markedly across settings:

Sometimes we are already involved with the patient before the hospitalisation, and still nobody informs us about the discharge. Then suddenly the patient is back home and we are expected to continue care, but without having received clear information about what was done in hospital or what exactly we are supposed to do. (Home-care nurse)

When APNs or liaison nurses were present, transitions were described as smoother and more predictable. These roles were associated with earlier communication, clearer preparation for families, and quicker resolution of uncertainties:

It truly depends on the ward. In some wards there is an advanced practice nurse who coordinates the discharge and keeps in contact with us, and there things usually work quite well. In other wards there is no one like that at all, and then it becomes much more complicated. Therefore, in the end it truly depends on where the patient is hospitalised. (Home-care nurse)

However, these roles were unevenly distributed across wards and organisations. In their absence, families again filled coordination gaps, a role that was often enacted through vigilance and corrective action when inconsistencies emerged in discharge documentation. Professionals expressed discomfort about this reliance, noting that such tasks exceeded what could reasonably be expected of families.

When there is no one clearly coordinating the discharge, very often the family becomes the point of reference. They are the ones who pass on information, who call the GP or the home-care service, and who try to organise what happens next. However, this puts a lot on them, because these are things that, in principle, should be organised by the system. (Hospital professional)

This theme illustrates how continuity often depended on whether specific individuals stepped in to bridge coordination gaps, because coordination was not embedded consistently across wards or services.

#### Theme 4: Families compensating for coordination gaps

Across stakeholder groups, families emerged as a primary source of compensatory work sustaining continuity after discharge. This theme draws predominantly on professional descriptions of family roles, supplemented by accounts from one family caregiver and one patient. The caregiver described coordinating services, managing medications, supervising safety, and interpreting clinical instructions, often at considerable emotional and physical cost:

I slept less, I lost weight, and even when I went out of the house to run an errand, I was always a bit worried, because I kept thinking: what might be happening at home? (…) It wasn’t that they left me completely on my own, no. However, one thing is when things are explained to you, and another thing is when you actually have to deal with them (home care providers) at home. (Family caregiver)

Similarly, the patient described considerable dependence on family members for safety and reassurance:

On my own, I wouldn’t have managed. My son was always there. He helped me with everything. (…) I wouldn’t have been able to handle everything at home by myself. (Patient)

Family involvement often compensated for incomplete information or late discharge notification, but it also generated emotional and physical strain. Providers described families as advocates who identified discrepancies and facilitated communication. However, reliance on family involvement sometimes generated tensions or unmet needs, especially when home environments required rapid adaptation or when caregiver capacity was limited. As one hospital professional observed, families were frequently expected to sustain continuity that the formal system had not reliably secured:

Very often this work is actually done by the family, by the caregiver, and this means an additional burden on the caregiver. In addition to caring for the patient, for their relative, they also manage all the professionals involved around the patient. Therefore, it is an extra burden (…) coordinating everyone and passing on the different pieces of information. (…) I had the opportunity to go and work at home with a colleague, and I saw that this is something that is done by the family. (Hospital professional)

Across accounts, family involvement was described as essential to maintaining continuity after discharge. However, the support available to caregivers was reported as variable and often insufficient for the demands placed on them.

#### Theme 5: Practices that supported smoother transitions

Participants identified practices that were associated with stronger continuity when coordination was anticipatory and responsibilities were clarified early. These were local practices observed under specific conditions. A central enabling practice was anticipation. Community providers described proactively contacting hospital wards by telephone when they anticipated that a discharge was approaching, typically based on estimated length of stay, informal signals from families, or prior experience with similar cases. This contact typically occurred before formal discharge confirmation and aimed to clarify the expected medication regimen, assess the need for home equipment, and coordinate family involvement:

Our strength is anticipation. If we start to suspect that a person might be discharged in the next few days, we already start calling the ward. (Home-care nurse)

This early engagement was described as allowing practical barriers to be addressed before the patient returned home, particularly with respect to medication access and family preparation.

Similarly, hospital professionals emphasised that transitions were described as smoother when there was time and dedicated capacity to act in advance:

When we manage to act a bit earlier, with time and dedicated competencies, this definitely helps during the transition phase. (Hospital professional)

At an organisational level, hospital leaders described how embedding anticipation into routine processes, such as defining a provisional discharge horizon early in admission, was intended to create the conditions for coordinated preparation across services:

As an internal objective, we have set ourselves a time limit, which is to establish a possible discharge date within 48 hours of admission to the ward. Within the first days, we should guarantee an analysis of the complexity of the case and of the possible fragilities, so that we can define a time horizon within which to organise everything. (Hospital leader)

Participants also highlighted the uneven distribution of such practices across wards. When bridging roles or proactive coordination structures were present, transitions were described as more orderly and predictable:

It depends a lot on the ward. In some wards it works better because there is someone who coordinates things earlier. (Home-care nurse)

Hospital leaders reinforced this observation, noting that dedicated coordination roles were perceived to have helped reduce fragmentation by centralising responsibility and clarifying information flows:

We saw that having a figure of reference who could gather all this information and act as an intermediary between the acute setting and the home truly made a difference. (Hospital leader).

Participants noted, however, that such practices were unevenly distributed across wards and organisations, that their presence depended on local structures.

### Analytic synthesis

Across themes, participants described hospital-home transitions as shaped by linked organisational conditions. Gaps in communication appeared to contribute to medication uncertainty, which in turn intensified coordination demands for professionals and families.

**Fig. 2:**
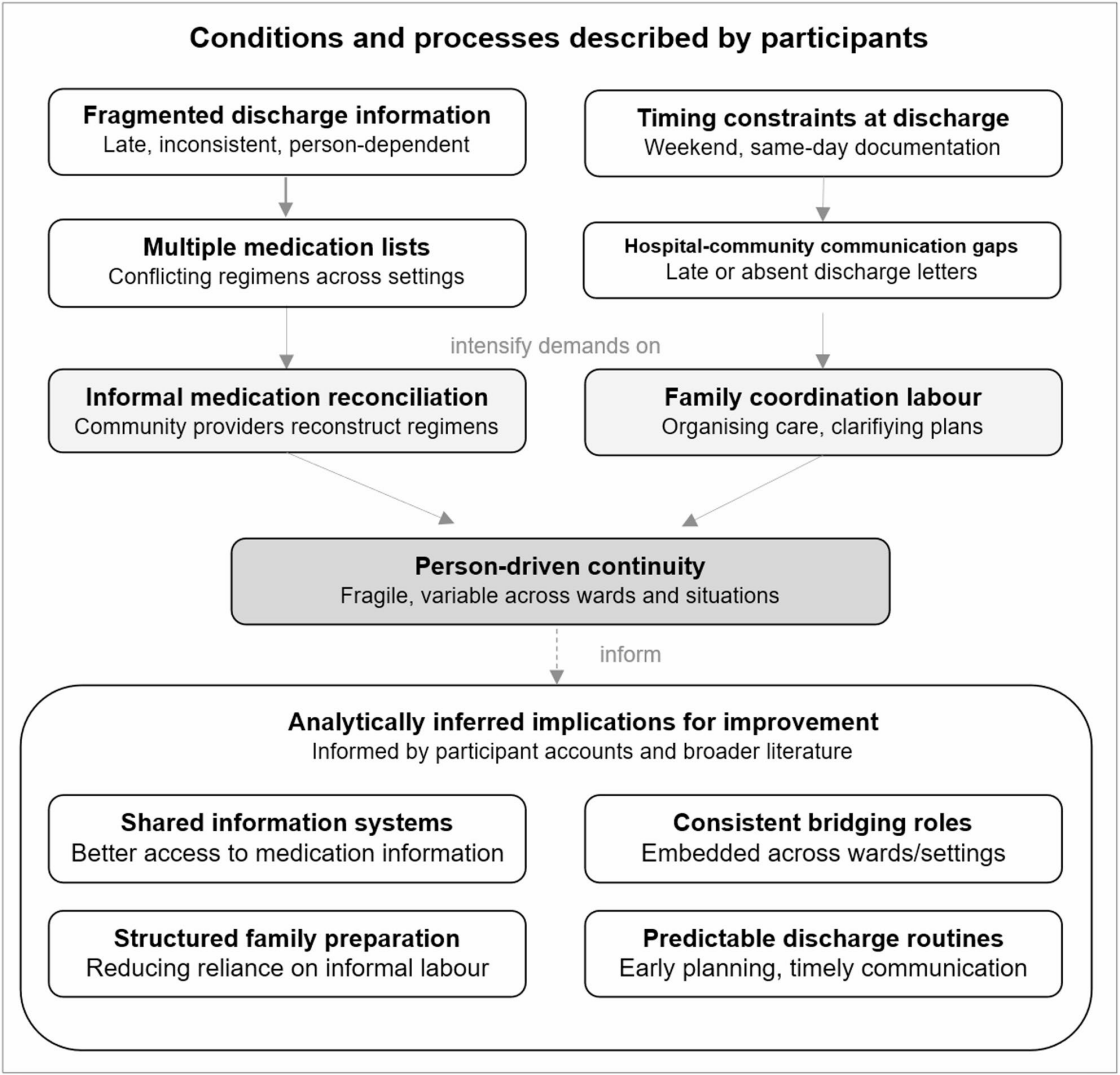
Analytic synthesis of how structural conditions and coordination processes featured in participants’ accounts of hospital-home transitions. Elements in the upper portion represent conditions and processes described by participants. Elements in the lower portion represent analytically inferred implications for improvement based on our analysis, informed by participant accounts and the broader literature. The synthesis was guided by Transitions theory, particularly the sensitising concepts of preparation (reflected in accounts of discharge timing and family preparation), role clarity evident in descriptions of communication gaps and bridging roles, and contextual conditions, illustrated by fragmented information flows and multiple medication lists

Figure 2 illustrates these interrelationships, depicting how decentralised system arrangements were described as affecting day-to-day coordination and stakeholder experiences of the hospital-home transitions.

## Discussion

This study explored how hospital-home transitions for older adults are experienced by hospital professionals, community-based providers, patients, and family caregivers in a decentralised Swiss canton. Participants described transitions as generally workable but often fragile. Continuity of care was often maintained through additional coordination work by professionals and family caregivers. Similar problems have been reported in other health system contexts. Evidence from Québec, Canada, suggests that formal integration does not automatically resolve coordination problems at the clinical interface, particularly when information sharing across providers remains limited [42, 43]. Studies from the UK likewise describe communication failures, missing or insufficient discharge information, and additional effort by receiving providers to secure the information needed during hospital-home transitions [44]. Fragmentation is therefore not unique to decentralised governance [45, 46]. In this setting, however, fragmentation was distributed across a broad, heterogeneous set of autonomous providers, and no single organisation held consistent oversight across settings. Responsibility was diffuse, which may help explain why familiar coordination problems took such a scattered form in this canton [43, 47, 48].

Participants described continuity as being sustained through ongoing work during discharge, particularly when information needed to be clarified or aligned. We refer to this pattern as person-driven continuity. In this study, it took three main forms. The first was anticipatory work, for example when community teams contacted wards before discharge was formally confirmed to prepare medications or family arrangements. The second was corrective work, such as reconciling conflicting medication lists and clarifying contradictory discharge documentation or follow-up plans after the patient had already returned home. The third was relational work, especially by families, who used practical knowledge and personal contacts to maintain continuity when formal pathways were unclear. In continuity-of-care terms, these efforts helped preserve informational and relational continuity when the formal arrangements had not secured them in advance [4, 9, 16, 49]. This pattern also helps explain why transitional experiences varied across wards and settings. Where APNs or liaison roles were present, participants described earlier communication, better preparation, and clearer allocation of responsibility. Where such roles were absent, continuity depended more heavily on who noticed a problem and took it on. Transitional quality was therefore more vulnerable to local arrangements and individual initiative in those contexts [19, 50–53].

Transitions theory was useful as a sensitising framework because it directed attention to preparation, role clarity, and contextual conditions [11, 54]. These concepts mapped closely onto the situations described by participants. Limited preparation time, especially when discharge documentation was completed late or discharge took place close to weekends, increased uncertainty and appeared to shift work downstream to families and community providers [1, 2, 44]. Role ambiguity was equally salient. At the boundary between hospital and home, participants were often left to decide for themselves what they were expected to do next and when to intervene [8, 49]. These conditions may help explain why discharge was experienced as more than a mere administrative handover. Safety was perceived to depend on whether people felt prepared and whether responsibility had been made clear [47]. At the same time, the findings also show the limits of Transitions theory on its own. The theory is better suited to capturing lived experience and interpersonal process than to informing organisational design or system governance. Integrated care and implementation science offer useful complements, as they draw attention to the conditions that make coordination possible across organisations, including governance arrangements, accountability structures, and shared information systems [24, 25, 48]. A related critique is that a mainly person-centred view may make the compensatory work of professionals and families seem routine, while drawing attention away from the organisational and governance conditions that make this work necessary. We therefore tried to keep both perspectives in view, understanding person-driven continuity as a sign that coordination has not been reliably established across providers.

Medication management made broader coordination difficulties especially visible. Where informational continuity was weak, the consequences became clinically visible through medicines. Community providers often had to reconstruct the final regimen after discharge, while GPs were left to interpret medication changes with limited context. Medication discrepancies appeared repeatedly in contexts when coordination between settings was weak. This aligns with wider literature showing that communication about medicines remains one of the most persistent vulnerabilities in care transitions for older adults [1–3, 16].

Although direct experiential data from patients and family caregivers were limited, the findings are consistent with evidence showing that hospital-home transitions often place substantial practical and emotional demands on families [5, 20, 21]. In this dataset, that pattern was described mainly by professionals, but it was also visible in the caregiver’s account of vigilance, exhaustion, and constant worry, and in the patient’s account of dependence on family to remain safe at home. These observations should be interpreted cautiously given the very small number of patient and caregiver participants. Even so, they point to a pattern worth noting: family involvement was often described as essential to continuity, but support for caregivers was reported as uneven and loosely structured. Families were described as having absorbed uncertainty that had not been resolved within the formal care process. In doing so, they were doing more than providing support. They were also helping to bridge coordination gaps that the formal system had not reliably addressed [5, 20, 21].

A further issue concerned the gap between awareness and implementation. Hospital leaders were able to describe strategies considered likely to support safer transitions, including early identification of a provisional discharge date and the use of designated coordination roles. However, these practices were not consistently embedded across wards, reflecting a gap between identified solutions and routine implementation [24, 25]. One plausible explanation consistent with implementation science is that, in decentralised systems, organisations may recognise the value of coordination between hospital and community without having the mandate or shared systems needed to implement it reliably across provider boundaries [24, 25]. Limited staffing, time pressure, and the demands of everyday ward routines may also have made consistent implementation difficult. As implementation science notes, recognising a problem and identifying a possible solution are rarely sufficient on their own [55–57]. Sustained uptake also depends on organisational readiness, leadership support, fit with existing workflows, and enabling policy conditions. The implementation gap observed here therefore appears structural [24, 25, 57].

The findings point to a small set of recurring conditions under which transitions were described as more orderly: timely discharge communication, earlier clarification and transmission of medication changes, explicit notification of community providers, and named roles for coordination. These are familiar features of transitional care models. The present study illustrates why they may be particularly relevant in mixed-provider settings where responsibility is easily dispersed [50–52, 58]. Communication was identified as particularly important, given that delays and inconsistent formats directly affected what happened after discharge [1, 3, 50]. Some participants also noted that communication difficulties were not confined to the discharge direction. Community providers described situations in which information about patients’ home context, existing support, and prior level of functioning was not clearly exchanged or taken into account when hospitalisation occurred. The difficulties described at discharge may therefore reflect a broader weakness in information sharing across the hospital-community interface more generally [15, 16], pointing to the need for coordination that functions across both directions.

### Limitations and strengths

This study has several limitations. Only one patient and one family caregiver participated. Their accounts offered valuable experiential insight, but they cannot balance the predominantly professional orientation of the dataset. This limited inclusion reflects recruitment difficulties during the immediate post-discharge period and should be kept in mind when interpreting findings relating to patient and caregiver experience. Similarly, although some participants referred to information gaps in the home-to-hospital direction, this was not a primary focus of data collection and arose only incidentally in a small number of accounts. The data therefore do not allow for conclusions about specific practices such as GP referral communication or the role of home-care nurses in this direction. The study was conducted in a single Swiss canton, and organisational arrangements may differ in other regions and health systems. At the same time, the detailed contextual description provided analytic transferability by allowing readers to judge the relevance of the findings in their own settings. Data density also differed across stakeholder groups, with hospital perspectives collected through focus groups and community perspectives through individual interviews. This difference may have shaped how experiences were voiced and framed, although triangulation across participant groups and data sources helped mitigate this potential source of bias. Finally, the cross-sectional design captured how transitions were understood at one point in time and does not allow for examination of how perceptions of continuity, burden, or responsibility may have changed across the postdischarge period.

A key strength of the study is the inclusion of diverse hospital and community perspectives across organisational boundaries in a decentralised care system. That breadth allowed the analysis to move beyond a single professional viewpoint and to examine how fragmentation was experienced and managed across the transition as a whole. The use of reflexive thematic analysis also supported attention to both organisational processes and lived experiences, and structured reflexive team discussions further strengthened interpretive rigour.

### Implications for practice and future research

The findings point to several areas worthy of attention for practice in decentralised settings. More predictable discharge communication, with timely transfer of final medication information and explicit notification of relevant community providers, was a recurring theme across stakeholder accounts. Medication management emerged as a particular concern across stakeholder accounts, since participants repeatedly described having to reconcile delayed or conflicting medication changes under time pressure. Participants also highlighted the value of APNs and liaison roles as identifiable points of coordination. Family caregivers likewise need clearer preparation for what will happen at home. These observations suggest several potential targets for quality improvement and evaluation: the timeliness of discharge information, the completeness and clarity of medication documentation, the notification of community providers, and the structured involvement of family caregivers.

Several directions for future research emerge from these findings. Larger studies involving patients and family caregivers, particularly longitudinal studies following patients through the post-discharge period, could deepen understanding of changes in transitional experience, caregiver burden, and perceived safety over time. Comparative research on decentralised and centralised health systems could further clarify which forms of fragmentation are linked to governance and which persist across system structures, building on earlier work from Québec and the United Kingdom. The findings also point to the value of evaluating APNs and liaison roles in mixed-provider settings, particularly to examine the conditions under which these roles may reduce reliance on individual effort and support continuity over time. Implementation research could usefully explore how practices such as early discharge planning, structured medication reconciliation, and proactive notification of community providers become embedded across wards and organisations in decentralised settings. The findings also highlight the need for more research on two-way communication across the hospital-community interface, including how information about patients’ home context, existing support, and functional status is shared at the point of hospitalisation.

## Conclusions

Participants described hospital-home transitions in this decentralised Swiss setting as highly variable, particularly with respect to communication, medication information, and how coordination responsibilities were understood. Continuity was often maintained through the informal coordination efforts of professionals and families. When coordination was weak, discharge processes became a source of potential risk for patients. Earlier planning, clearer communication, more robust medication information processes, and designated bridging roles represent areas that may reduce reliance on improvisation and support continuity that is less dependent on individual effort. The study provides an empirical basis for considering how transitional care processes might be developed and strengthened in ways that are sensitive to decentralised systems and local service arrangements.

## Electronic supplementary material

Below is the link to the electronic supplementary material.

Supplementary Material 1

## Data Availability

The datasets generated and/or analysed during the current study (anonymised interview transcripts and qualitative analysis files) are not publicly available owing to confidentiality considerations but are available from the corresponding author (LMS) on reasonable request and research purposes only.

## Abbreviations

APN(s): Advanced practice nurse(s)
COREQ: Consolidated Criteria for Reporting Qualitative Research
DNQP: German Network for Quality Development in Nursing (Deutsches Netzwerk für Qualitätsentwicklung in der Pflege)
EOC: Ente Ospedaliero Cantonale
FG: Focus group
FTE: Full-time equivalent
GP(s): General practitioner(s)
MRC: Medical Research Council
NICE: National Institute for Health and Care Excellence
UK: United Kingdom
