# Supplementary material for: Stakeholder insights into hospital-home transitions for older adults in a decentralised health system: a qualitative study
Source: BMC Health Serv Res. 2026 May 21;26:985. doi: 10.1186/s12913-026-14662-4 (PMC13371599; doi:10.1186/s12913-026-14662-4)
Supplement: Supplementary file 1 — Supplementary Material 1 [file 12913_2026_14662_MOESM1_ESM.docx]

**Additional file 1. Topic guides for interviews and focus groups**

The following focus group and interview guides were used to explore experiences of hospital–home transitions for older adults.

Guides were originally developed in Italian, informed by international discharge standards (e.g. NICE NG27, DNQP), and used flexibly to allow participants to elaborate on issues most relevant to their role and experience. Question wording and sequencing were adapted during data collection to follow participants’ accounts.

Table 1: Topic domains

| **Participant group** | **Core topic domains** | **Illustrative questions** |
| --- | --- | --- |
| **Hospital leadership / coordination roles (focus groups)** | Organisation of discharge; coordination responsibilities; communication with community services; medication processes; system constraints | - How are hospital discharges for older patients organised here? - Where do coordination responsibilities become unclear? - What makes some transitions more predictable than others? |
| **Hospital professionals directly involved in discharge (focus groups)** | Everyday discharge work; information exchange; medication at discharge; family involvement; uncertainty | - Can you describe a typical discharge process? - What information is often missing or arrives late? - How do you manage uncertainty at discharge? |
| **General practitioners / case managers (interviews)** | Post-discharge continuity; communication with hospital; medication changes; accountability | - When and how do you learn about a patient’s discharge? - How are medication changes communicated to you? - Who do you see as responsible for coordination? |
| **Home-care providers (public, private, self-employed) (interviews)** | Preparation for discharge; quality of information; medication reconciliation; coordination work; family role | - What information do you receive when a patient returns home? - How do you manage discrepancies in medication lists? - When do you need to compensate for missing information? |
| **Patients (≥65 years, recently discharged)**  **(interviews)** | Experience of discharge; understanding of care plan; returning home; support needs | - How prepared did you feel to go home? - What was unclear after discharge? - What helped you manage at home? |
| **Family caregivers (interviews)** | Caregiver role; coordination work; information gaps; emotional burden; support needs | - How were you involved in the discharge process? - What did you have to organise yourself? - What was most challenging for you? |
